# Genome Sequence of the Pyrene- and Fluoranthene-Degrading Bacterium *Cycloclasticus* sp. Strain PY97M

**DOI:** 10.1128/genomeA.00536-13

**Published:** 2013-08-01

**Authors:** Zhisong Cui, Guangsu Xu, Qian Li, Wei Gao, Li Zheng

**Affiliations:** Marine Ecology Research Center, the First Institute of Oceanography, State Oceanic Administration of China, Qingdao, China

## Abstract

*Cycloclasticus* sp. strain PY97M was isolated from a phenanthrene-degrading consortium, enriched from Yellow Sea sediment of China. Here, we present the draft genome sequence of strain PY97M, which contains 2,359,509 bp with a G+C content of 41.92% and contains 2, 264 protein-coding genes and 40 tRNAs.

## GENOME ANNOUNCEMENT

The bacteria of the genus *Cycloclasticus* are recognized as typical representatives of the obligate hydrocarbonoclastic bacteria (OHCB), which play a significant role in the biological removal of petroleum aromatic hydrocarbons from polluted marine environments (1, 2). *Cycloclasticus* sp. strain PY97M (CGMCC no. 4339) was isolated from Yellow Sea sediment of China (latitude, 36.67; longitude, 121.99; depth, 17.8 m) for its ability to use polycyclic aromatic hydrocarbons (PAHs) as the sole carbon and energy source. Although the *Cycloclasticus* strains exhibited high 16S rRNA similarity with each other, *Cycloclasticus* sp. PY97M distinguished itself from its counterparts by novel characteristics, including the ability to degrade the high-molecular-weight polycyclic aromatic hydrocarbons (HMW PAHs) pyrene and fluoranthene (3–6). The complete genome sequence of a pyrene-degrading bacterium *Cycloclasticus* sp. P1 (GenBank accession number CP003230) has already been published; nevertheless, a genome sequence of the pyrene- and fluoranthene-degrading species *Cycloclasticus* sp. PY97M could aid in the understanding of the *Cycloclasticus* species’ biodiversity and capacity for HMW PAH biodegradation in marine environments.

The genome sequence of *Cycloclasticus* sp. PY97M was determined by Shanghai Majorbio Bio-pharm Technology Co., Ltd. (Shanghai, China) using Solexa paired-end sequencing technology. A total of 12,847,820 paired-end reads (170-bp and 800-bp libraries) were generated to reach a 506-fold depth of coverage with an Illumina HiSeq 2000 (Illumina Inc., San Diego, CA). The reads were assembled using SOAPdenovo (ver. 1.05) (7, 8). The resulting genome sequence of *Cycloclasticus* sp. PY97M consists of 39 contigs (N90, 100,660) of 2,359,509 bp and had an average G+C content of 41.92%. Gene annotation was carried out by the NCBI Prokaryotic Genomes Automatic Annotation Pipeline (PGAAP) (http://www.ncbi.nlm.nih.gov/genomes/static/Pipeline.html), which was followed by manual editing. The genome contains 2,264 candidate protein-encoding genes (with an average size of 956 bp), giving a coding intensity of 91.60%. A total of 1,831 proteins could be assigned to cluster of orthologous groups (COG) families. Forty tRNA genes for all 20 amino acids and one 16S-23S-5S rRNA operon were identified by tRNAscan (ver. 1.23) (9) and rRNAmmer (ver. 1.2) (10).

We particularly analyzed the genes possibly responsible for PAH degradation; 12 genes encoding the alpha subunit of ring-hydroxylating dioxygenase were found in the genome of *Cycloclasticus* sp. PY97M. Moreover, seven genes encoding the beta subunit of ring-hydroxylating dioxygenase were also found in the genome sequence. The PY97M genome sequence and its curated annotation are important assets to facilitate better understanding of the physiology and metabolic potential of *Cycloclasticus* spp. and will open up new opportunities in the functional genomics of this species.

### Nucleotide sequence accession number. 

The nucleotide sequence comprising the *Cycloclasticus* sp. PY97M genome was deposited at DDBJ/EMBL/GenBank under the accession number ASHL00000000 (chromosome). The version described in this paper is the first version, ASHL00000000.
